# Thyroid Imaging Reporting and Data Systems: Applicability of the “Taller than Wide” Criterium in Primary/Secondary Care Units and the Role of Thyroid Scintigraphy

**DOI:** 10.3390/jcm13020514

**Published:** 2024-01-17

**Authors:** Manuela Petersen, Simone A. Schenke, Franziska Veit, Rainer Görges, Philipp Seifert, Michael Zimny, Roland S. Croner, Michael C. Kreissl, Alexander R. Stahl

**Affiliations:** 1 Department of General, Visceral, Vascular and Transplant Surgery, University Hospital Magdeburg, 39120 Magdeburg, Germany; 2 Department and Institute of Nuclear Medicine, Hospital Bayreuth, 95445 Bayreuth, Germany; 3 Division of Nuclear Medicine, Department of Radiology and Nuclear Medicine, University Hospital Magdeburg, 39120 Magdeburg, Germany; 4 Institute of Radiology Dr. von Essen, 56068 Koblenz, Germany; 5 Clinic for Nuclear Medicine, University Hospital Essen, 45147 Essen, Germany; 6 Clinic of Nuclear Medicine, University Hospital Jena, 07747 Jena, Germany; 7 Institute for Nuclear Medicine Hanau, 63450 Hanau, Germany; 8 Research Campus STIMULATE, Otto-von-Guericke University, 39106 Magdeburg, Germany; 9 Institute for Radiology and Nuclear Medicine, Radiologie im Zentrum (RIZ), 86150 Augsburg, Germany

**Keywords:** thyroid nodule, taller than wide, thyroid scintigraphy, risk of malignancy, fine needle biopsy, Thyroid Imaging Reporting and Data Systems

## Abstract

Background: To examine the applicability of the “taller than wide” (ttw) criterium for risk assessment of thyroid nodules (TNs) in primary/secondary care units and the role of thyroid scintigraphy therein. Methods: German bicenter study performed in a setting of primary/secondary care. Patient recruitment and analysis in center A was conducted in a prospective manner. In center B, patient data were retrieved from a database that was originally generated by prospective data collection. TNs were assessed by ultrasound and thyroid scans, mostly fine needle biopsy and occasionally surgery and others. In center A, only patients who presented for the first time were included. The inclusion criterion was any TN ≥ 10 mm that had at least the following two sonographic risk features: solidity and a ttw shape. In center B, consecutive patients who had at least ttw and hypofunctioning nodules ≥ 10 mm were retrieved from the above-mentioned database. The risk of malignancy was determined according to a mixed reference standard and compared with literature data. Results: In center A, 223 patients with 259 TNs were included into the study. For further analysis, 200 nodules with a reference standard were available. The overall malignancy rate was 2.5% (upper limit of the 95% CI: 5.1%). After the exclusion of scintigraphically hyperfunctioning nodules, the malignancy rate increased slightly to 2.8% (upper limit of the 95% CI: 5.7%). Malignant nodules exhibited sonographic risk features additional to solidity and ttw shape more often than benign ones. In addition to the exclusion of hyperfunctioning nodules, when considering only nodules without additional US risk features, i.e., exclusively solid and ttw-nodules, the malignancy rate decreased to 0.9% (upper limit 95% CI: 3.7%). In center B, from 58 patients, 58 ttw and hypofunctioning TNs on thyroid scans with a reference standard were available. Malignant nodules from center B were always solid and hypoechoic. The overall malignancy rate of hypofunctioning and ttw nodules was 21%, with the lower limit of the 95% CI (one-sided) being 12%. Conclusions: In primary/secondary care units, the lowest TIRADS categories for indicating FNB, e.g., applying one out of five sonographic risk features, may not be appropriate owing to the much lower a priori malignancy risk in TNs compared to tertiary/quaternary care units. Even the combination of two sonographic risk features, “solidity” and “ttw”, may only be appropriate in a limited fashion. In contrast, the preselection of TNs according to hypofunctioning findings on thyroid scans clearly warranted FNB, even when applying only one sonographic risk criterion (“ttw”). For this reason, thyroid scans in TNs may not only be indicated to rule out hyperfunctioning nodules from FNB but also to rule in hypofunctioning ones.

## 1. Introduction

Ultrasound (US) is an accurate and commonly used method for the detection and characterization of thyroid nodules (TNs). Following the first description of a standardized approach to the sonographic risk assessment of TNs by Horvath in 2009 [1], Kwak et al. published an easy-to-adopt Thyroid Imaging Reporting and Data System (Kwak-TIRADS) to describe suspicious malignant features in 2011: solid composition, hypoechogenicity, an irregular/microlobulated margin, microcalcification, and a taller than wide (ttw) shape. Increasingly, these TIRADS criteria are being used to make the decision as to whether a fine needle biopsy (FNB) is warranted or not [2]. In Kwak-TIRADS, FNB is already recommended for nodules with one of these suspicious US features (TIRADS 4a). The risk of malignancy for category 4a is reported to be 3.3% [2].

Several international societies have published modified US-based risk stratification systems (RSSs, Thyroid Imaging Reporting and Data Systems [TIRADS]) based on US features and lesion size [3,4,5,6]. In 2015, the American Thyroid Association (ATA) proposed a pattern-based five-tier RSS [6]. In 2016, the Korean Thyroid Association/Korean Society of Thyroid Radiology (KTA/KSThR) announced a pattern-based RSS (K-TIRADS) [3]. Similar to K-TIRADS, the European Thyroid Association (ETA) in 2017 proposed a pattern-based five-tier RSS (EU-TIRADS) [4]. The American College of Radiology (ACR) published a scoring-based system (ACR TI-RADS) [5]. These RSSs include recommendations for FNB based on different categories and size thresholds. The risk of malignancy of TNs in the respective lowest categories for the recommendation of FNB appear to be similar in different systems. In the ATA guidelines, it is given as 5–10%, in K-TIRADS as 3–15%, in EU-TIRADS as 2–4%, and in ACR TI-RADS as 5% [3,4,5,6]. The task force of the KSThR revised K-TIRADS in 2021 and the risk of the lowest category of malignancy was reported to be lower than with K-TIRADS (3–10%) [7]. Thyroid scans are neglected in all RSSs, in contrast to routine clinical practice, at least in Germany.

TIRADS appears to work well in tertiary/quaternary care units; however, it is less clear if it is applicable to primary/secondary care units [8,9]. Previous studies from our group have shown that in primary/secondary care units, the ttw shape of TNs is a frequent finding with a rate of 17%. In such a “real-life” setting, ttw nodules were associated with a low malignancy rate—around 1%. Without other hints of malignancy, a ttw shape appears to be a normal growth pattern in nodules at the dorsal region of the thyroid or with contact with a posterior horn or posteroinferior horn, rather than a risk factor. In this regard, ttw growth complies with a pole concept of goiter growth [10]. Further studies by our group and others have shown that the solidity of a TN is a frequent finding in the majority of benign nodules [8,11,12].

The five sonographic features of solidity, hypoechogenicity, irregular/microlobulated margin, microcalcification, and ttw form the basis for most TIRADS, as mentioned above. These five criteria can be subdivided into rather sensitive (80–90%) but unspecific (around 50%) features (solidity and hypoechogenicity) and rather insensitive (30–60%) but specific features (80–90%; irregular/microlobulated margin, microcalcification, and ttw) [8,10].

The finding of a hypofunctioning nodule on scintigraphy—although not included in TIRADS—would fall into the rather sensitive but unspecific subgroup [8]. For our study, in center A, we chose a particular combination of a rather sensitive criterion—“solidity”–with a rather specific criterion—“ttw”—irrespective of further US criteria as a decision threshold for FNB, in accordance with most TIRADS systems and with our clinical routine. The ttw shape, in this study, was chosen above the other “insensitive but specific” US features (see above) because of its highest interobserver agreement of around 90% and thus reproducibility and transferability to different investigation sites [13]. The combination of solidity and ttw generally falls under the highest or last but one highest risk category in different TIRADS systems [2,4,5]. In center B, we also chose a combination of the rather sensitive (hypofunctioning nodules) and the rather specific ttw features for further analysis of malignancy in accordance with our clinical routine.

The aim of this investigation was to clarify if such combinations of criteria can be used as a proper indication for FNB at primary/secondary care units and to clarify the role of thyroid scans. To our knowledge, this is the first attempt to test TIRADS criteria in a primary/secondary setting.

## 2. Materials and Methods

### 2.1. Patients

Patients presented in the two primary/secondary care units (center A and center B) over a period of approximately two years from 2020 to 2022. 

Recorded data comprise institution site, age, gender, lesion size in three dimensions (cranial–caudal, ventral–dorsal, medial–lateral), US features, lesion functionality on thyroid scan, cytological findings according to the Bethesda System [14], and/or histopathological results.

### 2.2. Reference Standard

Cytological (FNB) or histopathological (surgery) diagnoses, scintigraphically hyperfunctioning TNs (benign), or a negative ^99m^Tc-methoxy-isobuty-isonitrile (MIBI) imaging (benign) were taken as reference standard, as well as follow-up and laboratory results in one single case of each. In cytology, lesions with Bethesda II classification were considered benign. Bethesda I and III to V were not used as a reference standard if not corroborated by histology. Patients without a reference standard were excluded from further analysis.

### 2.3. Nodules

TNs with a size ≥10 mm were included. In center A, consecutive patients who presented for the first time and had solid and ttw nodules, irrespective of further sonographic or scintigraphic criteria, were included. In center B, consecutive patients having ttw and hypofunctioning nodules were retrieved from a prospectively enrolled database. Any further procedure, such as thyroid scan or FNB, was part of the clinical routine assessment in center A and also in center B.

### 2.4. Ethics

The data collection was conducted according to the guidelines of the Declaration of Helsinki and approved by the Ethics Committee of the Medical Faculty of the University Hospital of Duisburg-Essen, Germany (protocol code: 16-7022-BO, 04-AUG-2016, date of approval: 4 August 2016).

### 2.5. Examinations

Thyroid US was performed by experienced examiners (RG and ARS). The following US devices were used:

Center A: GE P9 (Healthcare, Milwaukee, WI, USA) linear US probe 12.0 MHz 

Center B: Canon Xario 100 TUS-X100 (Canon Medical Systems GmbH, Neuss, Germany) linear US probe 5.0-14.0 MHz 

Esaote MyLab (Esaote SpA, Genova, Italy) 40 linear US probe 7.5/10.0/14.0 MHz

US images were acquired in transverse and sagittal orientations. The following US features were documented:

Composition: solid, <10%, 10–50%, 50–90%, >90% cystic, spongiform.

Echogenicity: (marked) hypoechoic, isoechoic, hyperechoic, anechoic (cystic).

Margin: smooth, macrolobulated, microlobulated, irregular, ill-defined, extrathyroidal extension.

Calcification/spot: none, comet tail, macrocalcification, rim calcification, rim calcification with small extrusive soft tissue component, microcalcification.

Shape: wider than tall (wtt), taller than wide (ttw), round.

In almost all patients, thyroid scintigraphy was performed. Thyroid scans were performed with:

Center A: Siemens Symbia, 70 MBq ^99m^Tc-pertechnetate, planar imaging at 15 min; p.i. over 5 min.

Center B: MIE SD-X37, 70 MBq ^99m^Tc-pertechnetate, planar imaging at 15–25 min; over 5 min. 

Scintigraphy was conducted according to the European guidelines using ^99m^Tc-pertechnetate [15]. Scintigraphically hyperfunctioning TNs were not biopsied but were considered to be benign [16]. MIBI imaging was used for the risk assessment of hypofunctioning TNs in a few cases. Because of its high negative predictive value (NPV >> 90%), nodules with a negative result on MIBI imaging were considered to be benign [17,18,19].

### 2.6. Statistical Analysis

Malignancy rates were calculated as well as one-sided 95% confidence intervals (95% CI) using a standard formula. Where appropriate, Chi square tests were performed using Yates’s correction.

## 3. Results

### 3.1. Center A

#### 3.1.1. Overall Characteristics of Patients and Nodules

A total of 223 patients with 259 TNs with a nodule size of at least 10 mm (minimum diameter) were included into the study. Patient characteristics are given in Table 1. The reference standard was unequivocal FNB in 133 nodules, surgery in 36 nodules, negative MIBI imaging in 31 nodules, short-term clinical course (thyroiditis De Quervain’s thyroiditis) in 1 case, and laboratory exams (parathyroid adenoma) in 1 case. In summary, there were 200 nodules with an available reference standard (Table 1). These 200 nodules were used for further analysis.

#### 3.1.2. Nodules with Available Reference Standard (*n* = 200)

The overall malignancy rate in these 200 nodules was 2.5% (upper limit of the 95% CI: 5.1%). After the exclusion of scintigraphically hyperfunctioning nodules, the malignancy rate increased slightly to 2.8% (upper limit of the 95% CI: 5.7%). Malignant nodules exhibited more additional US risk features than benign ones (*p* < 0.05). When, in addition to the exclusion of hyperfunctioning nodules, considering nodules without additional US risk features only, i.e., exclusively solid and ttw nodules, the malignancy rate decreased from 2.8% (upper limit of the 95% CI: 5.7%) to 0.9% (upper limit of the 95% CI: 3.7%) (Table 2).

All results were considered to be significant at *p* < 0.05.

The size, presence of further US features, and scintigraphy results of the malignant solid and ttw nodules are shown in Table 3.

### 3.2. Center B

In center B, 58 patients with 58 TNs were retrieved in a retrospective but still consecutive manner given that the nodules were ttw and hypofunctioning. These nodules were larger as compared to those in center A. In addition to being hypofunctional and ttw, malignant nodules were always solid and hypoechoic in center B. The reference standard was surgery in 39 nodules, FNB in 19 nodules, and both FNB and surgery in 26 nodules. Overall, malignancy rate of hypofunctioning and ttw nodules was 21%, with the lower limit of the 95% CI (one-sided) lying at 12% (Table 4).

**Figure 1:**
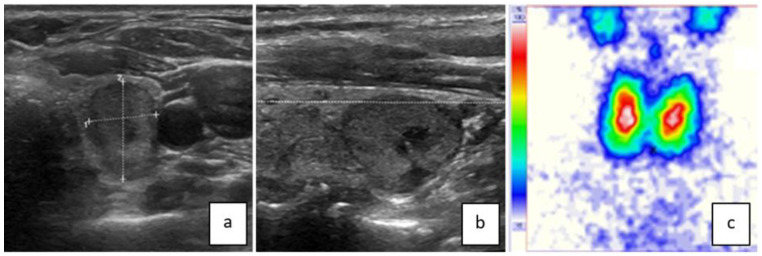
This ttw nodule in the left thyroid lobe has contact with the dorsal contour of the lobe ((**a**) = transversal/(**b**) = sagittal) complying with a pole concept of benign nodule growth [10]. The nodule is slightly hypoechoic with a smooth border, a small central cyst, and no calcifications. On the thyroid scan, the nodule is indifferent (**c**). The FNB result was between Bethesda II and III. Hemithyroidectomy revealed microfollicular adenoma.

**Figure 2:**
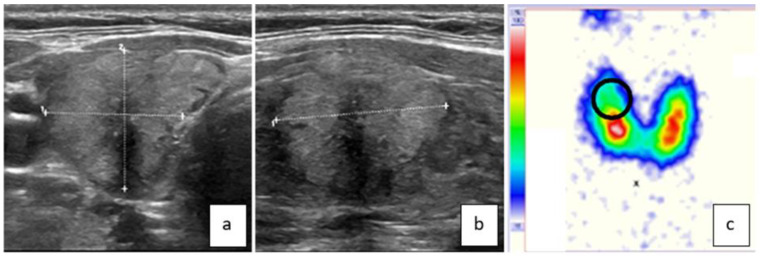
This ttw nodule in the right thyroid lobe has no direct contact with the dorsal contour and causes/follows no anatomic “horn” ((**a**) = transversal/(**b**) = sagittal) [10]. It is isoechoic, irregularly bordered, and has some central microcalcifications. Note also the central and dorsal acoustic attenuation of the US signal. The surrounding thyroid parenchyma is slightly hypoechoic because of an autoimmune thyroiditis. On the thyroid scan, the nodule is hypofunctioning (**c**). FNB showed follicular neoplasia and surgery revealed a papillary carcinoma with central sclerosis.

Figure 1 and Figure 2 show examples of ttw nodules.

## 4. Discussion

In a primary/secondary care setting, when applying solidity and ttw shape for preselection of TNs towards FNB (i.e., two out of five known US features), the reference standard yielded an overall malignancy rate of 2.5% (center A). This number is clearly below those in published data from different TIRADS systems where the combination of solidity and ttw generally falls under the highest or last but one highest risk category. For instance, for EU-TIRADS, the according malignancy risks are given as 26–87%, for ACR-TIRADS 5–20%, and for Kwak-TIRADS 7–38% [2,4,5].

The reason for this discrepancy can most likely be attributed to the different a priori risk for malignancy in primary/secondary care units compared to tertiary/quaternary units. Recently, a large-scale study in Germany has shown the a priori risk in a primary/secondary care units to be as low as 1% as compared to 10 to 20%, as given in TIRADS studies [2,6,16,20]. On an epidemiologic basis, the a priori risk (prevalence) for the malignancy of a TN may even be as low as one per mille [16].

There are good examples for the influence of preselection on the malignancy risk of TNs from the literature: a large meta-analysis included 32 studies with 15,641 TNs undergoing surgical intervention revealing 4166 malignant nodules and a rate of malignancy of 26.6.%. This rate decreased to 5.2% when all 80,079 nodules with FNB were considered [21]. Another meta-analysis included 8 published studies with 6362 operated nodules, with 2150 of these being malignant (33.7%). This rate decreased to 8.4% when all 25,445 nodules with FNB were considered [22].

The decision to perform FNB in TNs goes back to the positive predictive value (PPV) for malignancy for a given diagnostic criterion (or a combination of criteria) commonly being set at around 5% [2,3,4,5,6]. The PPV, however, is positively correlated with the a priori risk (prevalence) according to:

PPV = (sensitivity × prevalence)/[sensitivity × prevalence + (1 − specificity) × (1 − prevalence)](1)

For clinical situations with a low prevalence, such as in primary/secondary care units, the PPV becomes nearly directly proportional to the prevalence, according to:

PPV = prevalence × sensitivity/(1 − specifity)(2)

The more than ten times lower risk of malignancy of TNs in primary/secondary care units compared with study populations from tertiary/quatenerary care units thus explains well the ten times lower PPV for the combination of ttw and solidity found in our study compared to TIRADS.

Although not included in most TIRADS, it is routine clinical practice, at least in Germany, to rule out hyperfunctioning nodules before FNB since hyperfunctioning nodules harbor an extraordinarily low risk of malignancy, but may appear as follicular neoplasia on FNB [15,23]. Such an approach, in this study, allowed for the exclusion of more than 10% of solid and ttw nodule that, would otherwise have undergone futile FNB. A previous study showed that a relevant number of hyperfunctioning TNs show high-risk US features that would—if thyroid scans had not been used—have led to FNB, often with misleading results, i.e., follicular neoplasia [24].

Our study thus corroborates the recommendation in favor of thyroid scintigraphy before considering FNB. However, even after the exclusion of hyperfunctioning nodules, the resulting risk of malignancy was still relatively low (PPV 2.8%) compared to TIRADS, still questioning the need for FNB in solid and ttw nodules in primary/secondary care units.

When restricting the analysis to exclusively solid and ttw nodules, i.e., those without further US risk factors, including non-hyperfunctioning nodules only, the malignancy risk decreased to 0.9%. This is similar to the normal a priori risk encountered in primary/secondary care units and does not warrant FNB [16].

The recommendation of most TIRADS towards FNB in exclusively solid and ttw nodules is therefore at primary/secondary care units, which is not supported by our study. Adhering to TIRADS in such a setting would yield a relevant number of false-positive FNB results leading to futile thyroid surgery. Only in ACR TI-RADS, in exclusively solid and ttw nodules, FNB is restricted to nodules ≥15 mm, which would have spared futile FNB in 35/119 patients (i.e., ≈30%) in our study in center A [5,25]. However, even in the remaining 84 patients with exclusively solid and ttw nodules, FNB would not have been necessary owing to the still very low risk (<1%) of malignancy. In comparison, in a meta-analysis, 13,092 TNs were analyzed and the unnecessary FNB rates of different RSSs were investigated. The pooled unnecessary FNB rates of ACR TI-RADS, EU-TIRADS, ATA score, and K-TIRADS were 25%, 38%, 51%, and 55%, respectively [26]. The higher size threshold for FNB may explain the relative advantage of ACR TI-RADS over other RSSs in that meta-analysis.

In a recent university study, Grani et al. confirmed that the ttw shape is not an independent predictor of malignancy in TNs. In contrast, suspicious lymph nodes, extrathyroidal extension, irregular or infiltrating margins, marked hypoechogenicity, solid composition, and punctate hyperechoic foci (including microcalcifications and indeterminate foci) were documented as independent predictors of malignancy. Of the final study cohort of 903 nodules, 76 nodules (8.4%) were malignant. None of the TNs exclusively having a ttw shape as a sole sonographic risk factor turned out to be malignant [27].

It may be extrapolated from our study that, in primary/secondary care units, it is not appropriate to select candidates for FNB using only one single US criterion for malignancy, as indicated by many TIRADSs. Even some combinations (e.g., ttw and solidity, as in this study) may not suffice. For this reason, further preselection tools are sought in TNs in order to discern malignant nodules from benign nodules. In nuclear medicine divisions, there has long been the finding of a hypofunctioning nodule as such a preselection tool. In our study (center A) the addition of “hypofunctioning” of a nodule increased the PPV of ttw and solid nodules to about 4%. This value is still rather low and probably goes back to the small size of two (of five) carcinomas around one centimetre in center A. Bearing in mind this resolution limit of thyroid scintigraphy and taking into account the published malignancy risk for hypofunctioning nodules as 5% to 10%, the finding of a hypofunctioning and solid and ttw nodule should prompt FNB [23,28]. Radionuclide scans are the standard method for the evaluation of the function of TNs. In the future, it may be possible to determine the function of TNs also using US [29].

We tried to corroborate the value of a hypofunctioning nodule as a preselection tool for FNB with a small study from center B. In that part of our study, we substituted solidity with “hypofunctioning” by thyroid scintigraphy, in addition to ttw. A high PPV for malignancy (21%) was found for this combination, clearly warranting FNB. Interestingly, all carcinomas exhibited additional risk factors, namely solidity and hypoechogenity. The exclusive combination of ttw and hypofunctioning occurred in a small proportion of benign nodules (22%), only.

In comparison, a German multicenter study performed in primary/secondary as well as in tertiary/quatermary care units from the “German TIRADS Study Group” (GTSG) analysed 1211 hypofunctioning and indifferent TNs. The sensitivity (specificity) of solid composition, hypoechogenicity or marked hypoechogenicity, irregular or microlobulated shape, microcalcifications, and TTW for the detection of malignant TNs were 92% (21%), 85% (52%), 48% (92%), 55.0% (81.5%), and 33% (85%), respectively [8]. In that study population, over 80% of the nodules were hypofunctioning. The data showed a high sensitivity (75.1%) and a very low specificity (14.9%) for the feature of “hypofunctioning” for detecting malignant nodules. However, due to the exclusion of hyperfunctioning lesions and patient recruitment at university hospitals, these data may not directly compare to primary/secondary care units.

### Limitations

This study is in part (center B), a retrospective analysis. However, this limitation may not have a profound influence on the results because the accordant analyses rely on a prospectively built database. In addition, we conducted a bicenter study with a fully prospective approach in center A. However, in center B, patient recruitment was not restricted to their first presentation elevating the a priori risk of malignancy compared to center A. Due to this selection bias, center B may be considered as secondary/tertiary center of care rather than a primary/secondary center (center A). In addition, the fact that ttw and hypofunctioning nodules occurred in only 22% of benign nodules in center A and, conversely, there was a much higher surgery rate in center B (67% vs. 14% in center A) points to a bias towards more suspicious nodules in center B as compared to center A. This may explain the much higher PPV in hypofunctioning and ttw nodules at center B (21%) compared to center A (4%).

Finally, iodine deficiency is a well-known risk factor in the development of nodular thyroid disease and heavily affects a priori risks in TNs towards lower risks in iodine-deficient areas [30]. Center A is situated in the south of Germany with a long history of iodine deficiency, whereas center B is located in the middle of Germany.

## 5. Conclusions

In primary/secondary care units, the risk of malignancy for a TN with a given (combination of) US criteria—according to TIRADS—appears to be much lower than published on selected patient cohorts, i.e., at tertiary/quaternary care units. Therefore, it is necessary to preselect nodules in order to arrive at higher pre-test probabilities for malignancy that warrant FNB. Such a preselection may be accomplished by requesting more US risk factors for FNB than published. In addition, performing a thyroid scan may be helpful for TN above 10 mm, knowing that thyroid carcinomas are mostly hypofunctional, rarely indifferent. Consideration of these aspects may help to avoid unnecessary diagnostic and therapeutic procedures.

## Figures and Tables

**Table 1:** Characteristics of patients and nodules in center A.

Patients (*n* = 223)	Nodules ttw + Solid (*n* = 259)	Diagnostics	Reference Standard *n* = 200
female/male 144/79 age (years) 61 ± 14	max. size (mm) 20 ± 10	FNB: 149 scintigraphy: 252 MIBI scan: 8	unequivocal FNB: 133 surgery: 36 other (MIBI imaging, clinical course, laboratory values): 33

Abbreviations: FNB—fine needle biopsy; ttw—taller than wide; max—maximum; mm—millimeter; MIBI—^99m^Tc-methoxy-isobuty-isonitrile.

**Table 2:** Benign and malignant nodules with the gold standard in center A (*n* = 200): characteristics and risk of malignancy (ROM).

					After Exclusion of Hyperfunctioning Nodules (*n* = 176)
	Benign	Malignant	*p*	Benign	Malignant
number	195	5 →ROM 2.5% 95% CI: 5.1%		171	5 ROM 2.8% upper limit 95% CI: 5.7%
main size (mm)	20	23			
nodules ≥15 mm	127	3	0.81	113	3 (*p* = 0.085)
hypoechoic	47	1	0.75		
irregular margin	19	1	1		
microcalcification	23	4	<<0.01		
number additional US risk features per nodule	89/195	6/5	0.02		
nodules without additional US risk features, i.e., exclusively solid and ttw	131	1	0.09	119 (of these 84 ≥15 mm)	1 (*p* = 0.06) ROM 0.9% upper limit 95% CI: 3.7% (of these, 0 carcinomas ≥15 mm) ROM 0% upper limit 95% CI: 3.5%
scintigraphically					
hyperfunctioning	23	0	0.97		
hypofunctioning	47	2 →ROM 4% upper limit 95% CI: 9.0%	0.58		
indifferent	118	2	1		

Abbreviations: ROM—risk of malignancy; 95% CI—95% confidence interval; mm—millimeter, US—ultrasound.

**Table 3:** Characteristics of solid and ttw malignant nodules in center A (*n* = 5).

	#1	#2	#3	#4	#5
size (mm) transversal × sagittal × vertical	39 × 43 × 53	10 × 11 × 18	19 × 20 × 24	9 × 10 × 11	10 × 11 × 11
hypoechoic	no	yes	no	no	no
irregular margin/microlobulation	no	no	yes	no	no
microcalcification	yes	yes	yes	no	yes
scintigraphy	hypo-functioning	-	hypo-functioning	indifferent	indifferent

Abbreviations: mm—millimeter.

**Table 4:** Characteristics of benign and malignant nodules, risk of malignancy, and reference standard in center B (*n* = 58).

	Benign *n* (%)	Malignant *n* (%)	*p*		Reference Standard *n* (%)
number	46 (79)	12 (21)		→ROM 21% lower border 95% CI: 12%	FNB 45 (78)
mean size (mm)	29	25			surgery 39 (67)
≥15 mm	46 (79)	10 (17)	0.05		
hypoechoic	29 (50)	12 (21)	0.03		
irregular margin	6 (10)	7 (12)	0.003		
microcalcification	8 (14)	6 (10)	0.05		
solid	33 (57)	12 (21)	0.09		
hypofunctioning + ttw + solid *	10 (17)	0 (0)	0.18		

Abbreviations: ROM—risk of malignancy; 95% CI—confidence interval; FNB—fine needle biopsy; ttw—taller than wide; mm—millimeter. * exclusively, without additional US risk features. All results were considered to be significant with *p* < 0.05.

## Data Availability

The data that support the findings of this study are available from the corresponding author upon reasonable request.
